# Identification of Genetic Loci Affecting Flag Leaf Chlorophyll in Wheat Grown under Different Water Regimes

**DOI:** 10.3389/fgene.2022.832898

**Published:** 2022-03-15

**Authors:** Bin Yang, Xiaojie Wen, Hongwei Wen, Yanru Feng, Jiajia Zhao, Bangbang Wu, Xingwei Zheng, Chenkang Yang, Sanwei Yang, Ling Qiao, Jun Zheng

**Affiliations:** ^1^ Institute of Wheat Research, Shanxi Agricultural University/ State Key Laboratory of Sustainable Dryland Agriculture, Linfen, China; ^2^ College of Agricultural Economics and Management, Shanxi Agricultural University, Taiyuan, China; ^3^ Biotechnology Research Institute, Chinese Academy of Agricultural Sciences, Beijing, China; ^4^ Institute of Crop Science and Resource Conservation (INRES), Crop Science, University of Bonn, Bonn, Germany; ^5^ Department of Agronomy and Crop Physiology, Institute for Agronomy and Plant Breeding, Justus Liebig University Giessen, Giessen, Germany

**Keywords:** wheat, drought, chlorophyll, flag leaf, quantitative trait locus

## Abstract

Chlorophyll content of the flag leaf is an important trait for drought resistance in wheat under drought stress. Understanding the regulatory mechanism of flag leaf chlorophyll content could accelerate breeding for drought resistance. In this study, we constructed a recombinant inbred line (RIL) population from a cross of drought-sensitive variety DH118 and drought-resistant variety Jinmai 919, and analyzed the chlorophyll contents of flag leaves in six experimental locations/years using the Wheat90K single-nucleotide polymorphism array. A total of 29 quantitative trait loci (QTLs) controlling flag leaf chlorophyll were detected with contributions to phenotypic variation ranging from 4.67 to 23.25%. Twelve QTLs were detected under irrigated conditions and 18 were detected under dryland (drought) conditions. Most of the QTLs detected under the different water regimes were different. Four major QTLs (*Qchl.saw-3B.2*, *Qchl.saw-5A.2*, *Qchl.saw-5A.3*, and *Qchl.saw-5B.2*) were detected in the RIL population. *Qchl.saw-3B.2*, possibly more suitable for marker-assisted selection of genotypes adapted to irrigated conditions, was validated by a tightly linked kompetitive allele specific PCR (KASP) marker in a doubled haploid population derived from a different cross. *Qchl.saw-5A.3*, a novel stably expressed QTL, was detected in the dryland environments and explained up to 23.25% of the phenotypic variation, and has potential for marker-assisted breeding of genotypes adapted to dryland conditions. The stable and major QTLs identified here add valuable information for understanding the genetic mechanism underlying chlorophyll content and provide a basis for molecular marker–assisted breeding.

## Introduction

Chlorophyll is the key element for photosynthesis, which captures light energy to drive electron transfer to its reaction center. Chlorophyll content is positively correlated with photosynthetic efficiency (Avenson et al., 2005), directly affecting the accumulation of photosynthates (Guo et al., 2008; Zhang et al., 2009a). Under abiotic stress situations such as drought, high temperature, salinization, and heavy metal presence, genotypes with higher chlorophyll content maintain higher photosynthetic capacity that helps to maintain higher yield achievement (Vijayalakshmi et al., 2010; Kumar et al., 2012; Ilyas et al., 2014; Talukder et al., 2014; Awlachew et al., 2016; Gupta et al., 2020; Bhoite et al., 2021; Borjigin et al., 2021). Photosynthetic activity in the flag leaves of wheat contributes about 50% to the grain yield (Verma et al., 2004; Zhu et al., 2016). Drought stress at the grain-filling stage is a common occurrence in wheat crops. This leads to accelerated degradation of chlorophyll in photosynthetic organs such as leaves, reduced photosynthetic rate, and decreased photosynthetic efficiency (Yang B. et al., 2016), hence lower fixation and assimilation of CO_2_ (Yang D. et al., 2016) leading to restricted dry matter accumulation and grain development (Farooq et al., 2014). Therefore, the chlorophyll content in flag leaves is regarded as an indicator of drought resistance in wheat under drought stress (Farooq et al., 2014; Barakat et al., 2015). Molecular studies on the genetic regulation of flag leaf chlorophyll content are therefore of considerable significance for maintaining and improving yield potential under drought stress conditions.

Synthesis and degradation of chlorophyll is a complex biological process, which not only involves many genes and cellular metabolic pathways, but is also readily affected by internal and external environments. Quantitative trait locus (QTL) analysis and gene cloning following construction of high-density genetic linkage maps is an effective way to study the genetics of chlorophyll (Verma et al., 2004; Thomas and Ougham, 2014; Rasheed et al., 2020). In rice, more than 900 QTLs affecting chlorophyll content have been identified by QTL mapping (Ye, 2016). More than 120 leaf color–related genes have been cloned (Yang et al., 2020), among which 14 were involved in chlorophyll synthesis. These included *OsCAO1* encoding a chlorophyll oxygenase (Yang Y. et al., 2016); *OsCHLH*, *OsCHLD*, and *OsCHLI* encoding subunits of a magnesium-chelating enzyme (Jung et al., 2003; Zhou et al., 2012; Zhang et al., 2015); and *YGL1* encoding a chlorophyll synthase (Liu et al., 2016). In addition, eight genes related to stay green were cloned in rice, including a *DYE1*-encoded light capture complex I subunit (Yamatani et al., 2018), *EF8* encoding a HAP3 subunit of the HAP complex (Feng et al., 2014), and *SGR* that is involved in decomposition of chlorophyll (Morita et al., 2009). Some of these cloned genes have been successfully applied to rice breeding. Chen et al. (2020) found that overexpression of chloroplast gene *D1* increased rice biomass by 20.6–22.9% and yield of transgenic rice by 8.1–21.0% under field conditions. Thus, identification of major QTLs/genes related to chlorophyll synthesis and degradation in grain crops could have application in wheat breeding.

The wheat genome is about 17 Gb, 80–90% of which are highly repetitive sequences. Studies on the genetic mechanisms regulating chlorophyll lag behind those in model crops such as rice (Sultana et al., 2021). Moreover, the studies that have been reported involved different wheat populations and growth stages (Zhang et al., 2009a; Ilyas et al., 2014; Yu et al., 2014; Yang B. et al., 2016). The 82 reported QTLs controlling chlorophyll content were distributed across all 21 chromosomes (Quarrie et al., 2006; Zhang et al., 2009a; Zhang et al., 2009b; Kumar et al., 2010; Vijayalakshmi et al., 2010; Ilyas et al., 2014; Saleh et al., 2014; Barakat et al., 2015; Li et al., 2015; Yang D. et al., 2016; Gupta et al., 2017; Shi et al., 2017; Yan et al., 2020).

As chlorophyll content is affected by water availability and environmental conditions, there are few stably expressed major QTLs (Yang D. et al., 2016). Most studies involved widely dispersed SSR markers and there are no reports on the application of QTL for chlorophyll content in wheat breeding. A few major stay green QTLs have been fine-mapped (Li et al., 2018; Wang et al., 2020a; Gupta et al., 2020). For example, the F_2_ population involving early senescence mutant M114 with significantly reduced chlorophyll content in flag leaves was analyzed by BSR-Seq, and the *els1* gene was located in the *WGGB303–WGGB305* marker interval of 2BS, with 1.5 cM genetic distance (Li et al., 2018). Wang et al. (2020b) analyzed the inheritance of F_2_ population constructed with premature senescence mutant LF2099 and Chinese Spring, and mapped the *els2* gene to the marker interval of *2BIP09–2BIP14* on 2BL, and its genetic distance was 0.95 cM. There is no report on map-based cloning of genes regulating wheat chlorophyll content.

Genes *Tackx4*, *Tabas1-B1*, and *TaPPH-7A* contributing to chlorophyll content in wheat were identified by homologous cloning in wheat. Chang et al. (2015) cloned the *Tackx4* allele encoding a cytokinin oxidase on chromosome 3A and validated it using a Jing411 × Hongmangchun 21 RIL population. A major QTL co-segregating with *Tackx4* contributed 8.9–20.1% to chlorophyll content in four environments. Zhu et al. (2016) cloned *Tabas1-B1* encoding 2-cys peroxiredoxin BAS1 on chromosome 2B and identified a major co-segregating QTL that contributed 9.0–19.2% of the variation in chlorophyll content in three environments. Wang et al. (2019) cloned *TaPPH-7A* encoding a pheophorbide hydrolase on chromosome 7A and found that it was closely related to the chlorophyll content of flag leaves in plants grown under drought stress. However, none of these genes was validated by transgenesis. Clearly, synthesis and degradation of chlorophyll is a complex biological process involving many genes, but currently only a few major QTLs and genes related to chlorophyll content have been reported in wheat. Thus, different research approaches and populations to map QTL are of value for a better understanding of the genetics of chlorophyll content.

In this study, the chlorophyll content of flag leaves was analyzed by QTL analysis of a DH118 × Jinmai 919 RIL population grown in six environments with different moisture conditions and validated in a Jinchun 7 × Jinmai 919 DH population to 1) identify stable QTLs that regulate chlorophyll content in flag leaves and 2) study the effects of contrasting moisture availability on the QTLs with the objective of obtaining markers for wheat breeding.

## Materials and Methods

### Plant Materials and Plot Design

**FIGURE 1:**
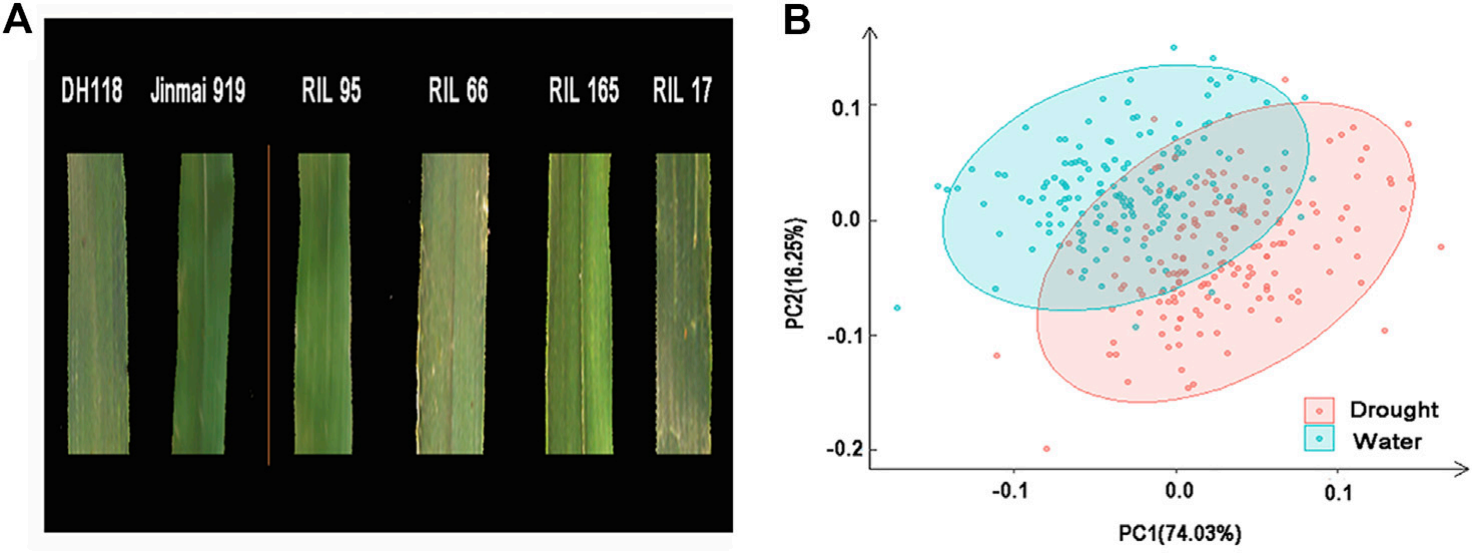
Phenotypic characteristic of the DJ population. **(A)** Phenotypes of the parents and selected RILs. **(B)** Principal components analysis (PCA) of chlorophyll content of flag leaves estimated for RILs grown under irrigated (water) and dryland (drought) conditions. Percentage variance accounted for by PC1 and PC2 is indicated in parentheses.

The populations with Jinmai 919 as a same parent included 165 F_10_ RILs from cross DH118 × Jinmai 919 (DJ) and 168 doubled haploid (DH) lines from Jinchun 7 × Jinmai 919 (JJ). DH118, a high-yielding variety selected for irrigated conditions by the Institute of Wheat Research, Shanxi Agricultural University, has dark green leaves and high chlorophyll content (Figure 1A). Jinmai 919 developed by the Institute of Wheat Research, Shanxi Agricultural University, has strong drought resistance, light green leaves, and good stay green characteristics (Figure 1A). Bred by the Institute of Maize Research, Shanxi Academy of Agricultural Sciences, Jinchun 7 is also a high-yielding variety for irrigated conditions. The DJ population was used for QTL mapping, and the JJ population was used for validating QTLs identified in the mapping population.

The DJ population was planted at Yaodu Experimental Station (36°08′N, 111°52′E, YD) and Hancun Experimental Station (36°25′N, 111°67′E, HC) at Linfen in Shanxi province in 2018–2019, 2019–2020, and 2020–2021. Plants were grown under irrigated and dryland (drought stressed) conditions in each year providing six environments designated as E1 (well-watered, 2019 YD), E2 (well-watered, 2020 YD), E3 (well-watered, 2021 YD), E4 (drought stressed, 2019 HC), E5 (drought stressed, 2020 HC), and E6 (drought stressed, 2021 HC). The JJ population was planted under the environmental conditions of E1, E2, E3, E4, and E5. The field design for both populations consisted of randomized complete blocks with three replications. Each plot consisted of two 1.5 m rows spaced 0.3 m apart at 21 seeds per row. After sowing, the Hancun site relied on natural precipitation during the whole growth period, 132 mm, 154 mm, and 147 mm in 2018–2019, 2019–2020, and 2020–2021, respectively; the Yaodu site was irrigated.

### Phenotypic Evaluation and Data Analysis

Ten plants flowering on the same day were randomly selected from each line at 10 days after flowering. The chlorophyll contents of flag leaves were measured using a SPAD-502 (Konica-Minolta, Japan) chlorophyll meter at 7:00 to 10:00 h. Each leaf was measured three times—at the base, mid-region, and tip—and the average value was used for analysis (Yang B. et al., 2016). Average values were also determined for each environment.

SPSS 21.0 software (SPSS, Chicago, IL, USA; http://www.spss.com) was used to perform Student’s *t*-tests, correlation analysis, and ANOVA comparing phenotypic data from the two environments. SAS (SAS Institute, Cary, NC, USA; https://www.sas.com) was applied for calculating best linear unbiased predictions (BLUPs) and broad sense heritabilities (*H* ^*2*^).

### High-Density Genetic Linkage Map Construction and QTL Mapping

DNA was extracted from all RILs and DH lines and respective parents using the CTAB method (Vijayalakshmi et al., 2010). The RIL population was genotyped with the Infinium wheat SNP 90K iSelect assay (Illumina Inc., San Diego, CA, USA) developed by the International Wheat SNP Consortium (Wang et al., 2014). IciMapping v4.0 (https://www.isbreeding.net) was used to construct a high-density genetic linkage map (Li et al., 2021). SNP markers with no recombination were placed into a single bin using the “BIN” function in IciMapping V4.0. The final markers were chosen with a minimum percentage of missing data and sorted into different groups with LOD thresholds ≤8 by the “Grouping” function in JoinMap 4.0 (Li et al., 2021).

The QTLs were detected using WinQTLCart version 2.5 (https://brcwebportal.cos.ncsu.edu/qtlcart/WQTLCart.htm) based on the composite interval mapping method. QTLs were proclaimed significant at logarithm of odds (LOD) scores >2.5. The QTL contributing more than 10% to phenotypic variation in a certain environment (including BLUP) and detected in three environments (including BLUP) was considered as a stable and major QTL. QTLs less than 1 cM apart or sharing common flanking markers were treated as a single locus. The QTLs were named according to McCouch et al. (1997). The closest marker sequences flanking QTL were compared with the Chinese Spring reference genome sequence in the wheat multiomics website database (http://wheatomics.sdau.edu.cn/jbrowse-1.12.3-release/?data=Chinese_Spring1.0) to determine the physical locations of the QTL.

### Marker Development and Validation of Major QTLs

To develop kompetitive allele specific PCR (KASP) tags from the peak marker SNP sequence of the major QTLs, two specific primers (F1/F2) and a universal primer (R) were designed for each SNP. An F1 tail that could bind to induce FAM fluorescence and an F2 tail that could bind to induce HEX fluorescence were added to the specific sequences. KASP primers were designed by Polymarker (http://www.polymarker.info/) and synthesized by Beijing Jiacheng Biotechnology Co., Ltd. (Supplementary Table S1). The developed KASP markers were used in PCR to detect previously identified QTLs in the JJ population as a means of validation. Following genotyping, the validation population was divided into two groups and differences in chlorophyll content of flag leaves between the groups were assessed by *t*-tests in SAS V8.0.

### Gene Prediction Within QTL

Genes within the target region of major QTL were obtained using the genome browser (JBrowse) on the Triticeae Multi-omics website (http://wheatomics.sdau.edu.cn/). The GO (gene ontology) database and R package cluster profiler were applied for functional annotation and enrichment analysis of genes in the QTL regions. Identification of orthologs in wheat and rice was conducted using the Triticeae-Gene Tribe website (http://wheat.cau.edu.cn/TGT/). The expVIP public database (http://www.wheat-expression.com/) was used to search for expression data of genes in eight tissues and organs, perform log2 conversion processing, and analyze the expression patterns of candidate genes.

## Results

### Analysis of Phenotypic Data

The chlorophyll contents of flag leaves of DH118 and Jinmai 919 ranged from 52.16 to 60.22 and 48.80 to 58.42, respectively, across the six environments. The chlorophyll content of DH118 was consistently higher than that of Jinmai 919, and the difference was significant in E1 and E6 (*p* <0.05), and highly significant in E2 and E5 (*p* <0.01) (Table 1). The correlation of chlorophyll contents among different environments for the RIL population was highly significant (*p* <0.01), and correlation coefficients ranged from 0.303 to 0.711 (Supplementary Table S2). The *H* ^*2*^ of chlorophyll content was 0.90, indicating that chlorophyll content was largely determined by genetic factors. Principal component analysis showed that environmental factors had considerable influence on phenotypic values, and drought stress increases the phenotypic variation (Figure 1B). Chlorophyll content of the RIL population was mostly between the two parents under E2, E3, E4, E5, and E6 environments, showing a continuous distribution. Bidirectional transgressive segregation was also observed in chlorophyll content among the RIL population under E1 condition (Table 1).

**TABLE 1:** Chlorophyll contents in flag leaves in parents and RILs derived from cross DH118 × Jinmai 919 in six environments

Trait	Environment	DH118	Jinmai 919	Min	Max	Mean	SD	*H* ^*2*^
CHL	E1	52.16*	48.80	48.02	60.04	54.04	2.37	0.90
—	E2	55.40**	50.35	47.00	57.80	54.06	2.20	—
—	E3	54.33NS	53.20	47.50	59.12	54.27	2.37	—
—	BLUP—W	53.99*	51.41	48.95	57.99	54.12	1.59	—
—	E4	60.22NS	58.42	52.84	63.50	58.50	2.31	—
—	E5	57.00**	51.63	45.40	59.42	54.21	2.25	—
—	E6	57.45*	55.13	48.32	59.74	56.44	2.60	—
—	BLUP—D	57.88*	55.30	51.35	59.26	56.36	1.66	—
—	BLUP	56.01NS	53.14	49.86	58.97	55.24	1.65	—

H^2^, broad-sense heritability; BLUP—W, best linear unbiased prediction under irrigated conditions; BLUP—D, best linear unbiased prediction under dryland conditions; BLUP, best linear unbiased prediction; *, p <0.05; **, p <0.01; NS, not significant.

### Linkage Map Construction

A high-density genetic linkage map for the RIL population was constructed by using Wheat90k SNP chip. The total length of the map was 5,858.63 cM with an average genetic distance of 1.65 cM, including 3,553 SNP markers and covering all 21 chromosomes (Table 2). The numbers of SNP markers in the A, B, and D genomes were 1,395, 1,880, and 278, respectively, and the linkage lengths were 2,394.29, 2,953.31, and 511.03 cM, with average distances between markers of 1.72, 1.57, and 1.84 cM, respectively (Table 2). The D genome had the lowest marker coverage; the longest linkage group was 673.66 cM for chromosome 5B, and the shortest was 23.30 cM for chromosome 4D.

**TABLE 2:** Summary of linkage group and marker statistics obtained from a 90K SNP chip analysis of the DH118×Jinmai 919 RIL population

Chromosome	DH118 × Jinmai 919		
	No. of SNP markers	Length (cM)	Marker density (cM/marker)
1A	210	300.02	1.43
2A	166	315.04	1.90
3A	176	357.51	2.03
4A	144	272.00	1.89
5A	260	411.34	1.58
6A	245	352.09	1.44
7A	194	386.30	1.99
1B	363	557.69	1.54
2B	320	501.92	1.57
3B	279	445.65	1.60
4B	140	282.05	2.01
5B	391	673.66	1.72
6B	231	226.06	0.98
7B	156	266.29	1.71
1D	28	64.03	2.29
2D	87	138.15	1.59
3D	52	76.83	1.48
4D	9	23.30	2.59
5D	18	33.33	1.85
6D	61	100.43	1.65
7D	23	74.97	3.26
A genome	1,395	2,394.29	1.72
B genome	1,880	2,953.31	1.57
D genome	278	511.03	1.84
Total	3,553	5,858.63	1.65

### QTL Mapping for Chlorophyll Content under Different Environments

A total of 29 QTLs for chlorophyll content were detected on chromosomes 1B, 2A, 2B, 2D, 3A, 3B, 4B, 5A, 5B, 6B, 7A, and 7B. The LOD scores ranged from 2.58 to 10.70 and individual QTL explained 4.67–23.25% of the phenotypic variation in different environments (Table 3). Favorable alleles of 20 QTLs were derived from DH118 and favorable alleles of 9 QTLs were derived from Jinmai 919.

**TABLE 3:** Quantitative trait loci (QTL) for chlorophyll content detected in the DH118 × Jinmai 919 RIL population grown under different water regimes

QTL name	Environment	Chr	LOD	R^2^ (%)	Add	Left marker	Right marker	Genetic interval (cM)	Physical interval (Mb)
*Qchl.saw-1B*	E1	1B	2.58	6.34	0.61	*wsnp_Ex_c27176_36393952*	*Kukri_c25512_53*	417.616–434.069	640.848/648.454
*Qchl.saw-2A.1*	E5	2A	3.42	5.90	−0.56	*CAP11_s9154_121*	*BS00100472_51*	186.071–187.965	369.833/343.887
*Qchl.saw-2A.2*	E3	2A	5.13	10.51	−0.81	*Excalibur_c27023_134*	*RFL_Contig3071_626*	197.103–202.211	504.275/199.7962
*Qchl.saw-2B.1*	E5	2B	2.65	4.67	−0.50	*Ex_c19038_1581*	*Tdurum_contig20262_440*	43.902–47.744	19.394/18.932
—	BLUP	2B	2.74	5.24	−0.39	*Ex_c19038_1581*	*Tdurum_contig20262_440*	43.902–47.744	19.394/18.932
*Qchl.saw-2B.2*	E1	2B	3.60	7.79	−0.67	*Kukri_c34553_110*	*RAC875_c98387_145*	409.899–410.530	766.234/766.234
*Qchl.saw-2B.3*	E6	2B	4.84	9.83	−0.82	*RAC875_c19685_944*	*Ku_c2936_1987*	439.101–446.163	781.584/782.154
*Qchl.saw-2D.1*	E2	2D	3.41	6.90	0.59	*wsnp_Ex_rep_c68555_67394261*	*BS00018028_51*	6.997–12.707	344.298/145.396
*Qchl.saw-2D.2*	BLUP—W	2D	4.35	9.60	0.50	*BS00018028_51*	*Kukri_c22553_60*	12.707–18.956	145.396/108.922
*Qchl.saw-2D.3*	E1	2D	2.65	6.35	0.61	*Kukri_c22553_60*	*RAC875_c11911_431*	18.956–23.578	108.922/110.666
*Qchl.saw-3A.1*	E6	3A	3.08	5.63	−0.64	*RAC875_c20134_535*	*BobWhite_c37325_92*	48.634–61.187	14.851/20.328
*Qchl.saw-3A.2*	E3	3A	2.82	5.58	0.57	*BobWhite_s65081_93*	*Ra_c5515_2469*	171.493–173.675	510.690/514.112
*Qchl.saw-3B.1*	E2	3B	3.00	6.68	−0.58	*wsnp_Ex_c16569_25082817*	*Tdurum_contig31097_254*	21.883–28.083	817.822/811.448
*Qchl.saw-3B.2*	E1	3B	3.68	8.19	0.70	*BS00010818_51*	*Excalibur_c8284_580*	164.650–170.772	52.832/54.756
—	E3	3B	5.17	10.59	0.80	*BS00010818_51*	*Excalibur_c8284_580*	164.650–170.772	52.832/54.756
—	BLUP—W	3B	5.38	11.43	0.56	*BS00010818_51*	*Excalibur_c8284_580*	164.650–170.772	52.832/54.756
*Qchl.saw-4B.1*	E4	4B	3.08	5.76	0.57	*Excalibur_c17607_542*	*RAC875_c15872_141*	180.255–199.003	311.352/140.898
*Qchl.saw-4B.2*	E5	4B	4.13	7.94	0.63	*wsnp_Ex_c3119_5763762*	*wsnp_JD_c1549_2185341*	171.195–180.754	443.454/363.305
*Qchl.saw-4B.3*	BLUP—D	4B	5.26	11.33	0.56	*RAC875_c48283_1574*	*wsnp_Ex_c30695_39579408*	195.003–216.053	140.898/20.589
*Qchl.saw-5A.1*	E4	5A	3.23	6.24	0.84	*RAC875_rep_c109716_67*	*IACX448*	20.398–21.319	586.597/588.377
*Qchl.saw-5A.2*	E5	5A	2.79	5.33	0.71	*wsnp_Ra_c3414_6378271*	*Kukri_c61046_510*	23.831–26.668	582.387/569.547
—	BLUP—D	5A	3.11	5.28	0.53	*wsnp_Ra_c3414_6378271*	*Kukri_c61046_510*	23.831–26.668	582.387/569.547
—	BLUP	5A	5.44	10.76	0.77	*wsnp_Ra_c3414_6378271*	*Kukri_c61046_510*	23.831–26.668	582.387/569.547
*Qchl.saw-5A.3*	E2	5A	2.89	6.04	−0.55	*GENE-2735_151*	*RAC875_c79540_228*	52.132–57.368	586.598/615.305
—	E4	5A	7.58	15.62	−1.33	*GENE-2735_151*	*RAC875_c79540_228*	52.132–57.368	586.598/615.305
—	E5	5A	6.06	12.62	−1.05	*GENE-2735_151*	*RAC875_c79540_228*	52.132–57.368	586.598/615.305
—	E6	5A	7.26	14.10	−1.01	*GENE-2735_151*	*RAC875_c79540_228*	52.132–57.368	586.598/615.305
—	BLUP—D	5A	9.73	18.36	−0.95	*GENE-2735_151*	*RAC875_c79540_228*	52.132–57.368	586.598/615.305
—	BLUP	5A	10.70	23.25	−1.09	*GENE-2735_151*	*RAC875_c79540_228*	52.132–57.368	586.598/615.305
*Qchl.saw-5A.4*	E5	5A	2.84	4.94	−0.54	*wsnp_Ku_c7890_13514597*	*wsnp_Ex_c9842_16228523*	264.126–268.370	19.231/15.850
*Qchl.saw-5B.1*	E4	5B	4.22	8.28	0.67	*Excalibur_c5594_1051*	*BS00013829_51*	292.392–294.925	520.8872/526.397
*Qchl.saw-5B.2*	E5	5B	4.33	8.32	0.65	*RAC875_c32611_347*	*BS00093591_51*	320.986–322.563	536.681/536.052
—	BLUP	5B	3.77	7.45	0.46	*RAC875_c32611_347*	*BS00093591_51*	320.986–322.563	536.6813/536.052
—	BLUP—D	5B	6.86	12.15	0.59	*RAC875_c32611_347*	*BS00093591_51*	320.986–322.563	536.681/536.052
*Qchl.saw-6B.1*	E2	6B	3.36	7.62	0.62	*BS00064027_51*	*RFL_Contig2206_1694*	129.185–138.393	680.937/690.730
*Qchl.saw-6B.2*	E3	6B	4.76	9.70	0.76	*Tdurum_contig61383_627*	*Tdurum_contig42301_1583*	0.000–1.900	39.198/35.367
*Qchl.saw-7A.1*	E4	7A	3.21	6.21	0.60	*wsnp_Ex_c40247_47349166*	*BS00047691_51*	13.785–15.675	116.113/118.327
*Qchl.saw-7A.2*	BLUP—D	7A	5.07	8.74	0.51	*RAC875_c62204_772*	*BS00059928_51*	32.704–36.455	615.341/603.092
—	BLUP	7A	4.02	7.68	0.47	*RAC875_c62204_772*	*BS00059928_51*	32.704–36.455	615.341/603.092
*Qchl.saw-7A.3*	E5	7A	3.28	6.17	0.56	*CAP7_c4608_228*	*BS00105558_51*	65.630–71.807	540.857/587.912
*Qchl.saw-7A.4*	E6	7A	6.39	11.79	0.93	*BobWhite_c30461_131*	*Excalibur_c22219_254*	74.905–80.888	647.934/659.374
*Qchl.saw-7B.1*	E4	7B	2.99	5.77	0.60	*RAC875_rep_c81362_198*	*Excalibur_c29607_442*	81.331–82.605	231.32/244.279

Four major QTLs (*Qchl.saw-3B.2*, *Qchl.saw-5A.2*, *Qchl.saw-5A.3*, and *Qchl.saw-5B.2*) for chlorophyll content were detected on chromosomes 3B, 5A, and 5B, respectively. *Qchl.saw-5A.3* was detected in E2, E4, E5, E6, BLUP—D, and BLUP. The LOD values ranged from 2.89 to 10.70 and the QTL explained 6.04–23.25% of the phenotypic variation. The positive allele for *Qchl.saw-5A.3* was contributed by Jinmai 919 (Table 3). *Qchl.saw-3B.2* was detected in E1, E3, and BLUP—W, explaining 8.19–11.43% of the phenotypic variation. *Qchl.saw-5A.2* was detected in E5, BLUP—D, and BLUP, explaining 5.28–10.76% of the phenotypic variation (Table 3). *Qchl.saw-5B.2* was detected in E5, BLUP—D, and BLUP, and explained 7.45–12.15% of the phenotypic variation. The positive alleles for *Qchl.saw-3B.2*, *Qchl.saw-5A.2*, and *Qchl.saw-5B.2* were contributed by DH118 (Table 3).

### Additive Effects of the Major QTLs *Qchl.saw-3B.2*, *Qchl.saw-5A.2*, *Qchl.saw-5A.3*, and *Qchl.saw-5B.2* on Chlorophyll Content

**FIGURE 2:**
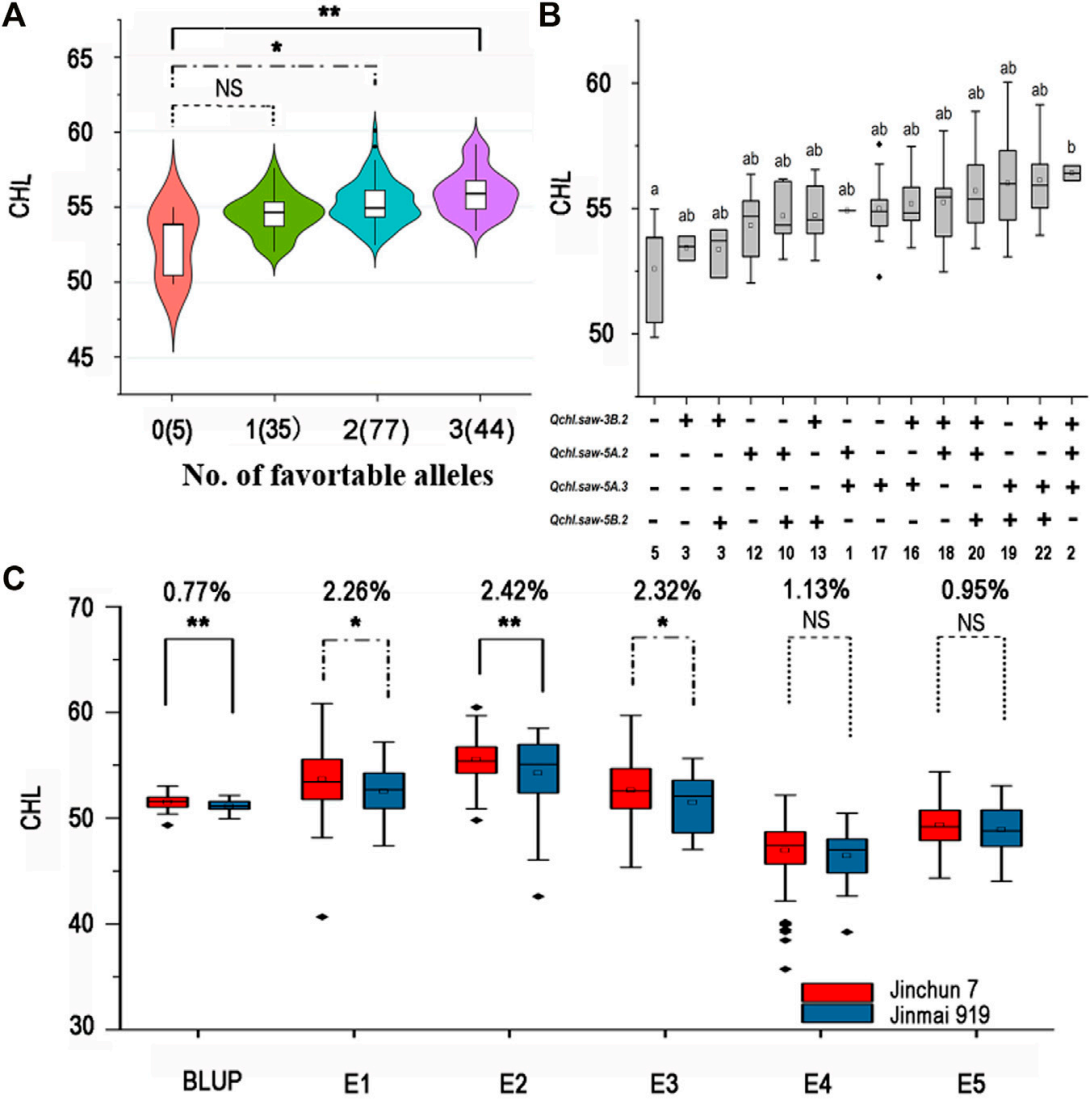
Additive effects and validation of major QTL. **(A)** Relationship of numbers of favorable alleles and chlorophyll content in the DJ population. **(B)** Linear regressions between the additive effects of QTL and chlorophyll content in the DJ population. Numbers of lines carrying the corresponding number of favorable alleles are shown in brackets. The letter above the bars indicates comparison results at the significant level 0.05 and respectively. “+” and “−” represent lines with and without the favorable alleles. **(C)** Validation of *Qchl.saw-3B.2* in JJ population. * and ** represent significance at *p* < 0.05 and *p* < 0.01, respectively. NS represents not significant.

Analysis of the additive effects of the four major QTLs showed that the number of favorable alleles increased chlorophyll content (Figure 2A, Supplementary Table S3). No RIL with all four favorable alleles was detected. The average chlorophyll content of RILs with three favorable alleles increased by 3.11–3.81 (5.91–7.24%) compared with RILs with no favorable allele. Among combinations, the average chlorophyll content of RILs with favorable alleles of *Qchl.saw-3B.2*, *Qchl.saw-5A.2*, and *Qchl.saw-5A.3* was the highest at 7.24% above that of lines with no favorable allele (Supplementary Table S3). In addition, the average chlorophyll content of lines with only *Qchl.saw-5A.3* allele in RIL population was higher than that of other lines with only one favorable allele (Figure 2B), indicating that the allele of *Qchl.saw-5A.3* had the highest genetic effect on chlorophyll content.

### Validation of the Major QTL *Qchl.saw-3B.2* in the JJ Population

To validate the four major QTLs, KASP markers for each QTL were used to evaluate their effects on chlorophyll content in the JJ population. The KASP markers for *Qchl.saw-5A.2* and *Qchl.saw-5A.3* were not polymorphic between Jinchun 7 and Jinmai 919. The effect of *Qchl.saw-5B.2* did not differ significantly between two contrasting phenotypic groups in JJ population. The effect of *Qchl.saw-3B.2* was significant (*p* <0.05) in E1 and E3, and highly significant (*p* <0.01) in E2 (Figure 2C). The chlorophyll content of lines with the favorable *Qchl.saw-3B.2* allele was higher than that without this allele, and the difference varied from 0.95 to 2.26% across environments.

### Candidate Genes in the Intervals of the Four Major QTLs

A total of 1,207 genes were identified in the four major QTLs; 73 genes in *Qchl.saw-3B.2* (52.83–54.76 Mb), 368 in *Qchl.saw-5A.2* (569.55–582.39 Mb), 735 in *Qchl.saw-5A.3* (586.59–615.30 Mb), and 31 in *Qchl.saw-5B.2* (536.05–536.68 Mb) (Table 3; Supplementary Table S4). According to gene functional annotations in the Gene Ontology (GO) public database, 174 of these genes are involved in chlorophyll metabolism and drought stress (Supplementary Table S5; Supplementary Figure S1). Analysis of gene expression in various tissues identified 18 candidate genes related to chlorophyll metabolism (Table 4).

**TABLE 4:** Functional annotation and enrichment of chlorophyll content QTLs on chromosomes 5A and 5B

Gene ID in wheat	Gene ID in rice	Gene symbol	Chr	Start (bp)	Stop (bp)	Ori	Function
*TraesCS5A02G377000*	—	—	chr5A	574,489,917	574,493,061	−	Integral component of membrane
*TraesCS5A02G382600*	*Os03g0742900*	*OsIAA13; OsIAA1*	chr5A	580,464,946	580,467,685	−	Auxin-activated signaling pathway
*TraesCS5A02G376700*	*Os03g0638800*	—	chr5A	574,343,360	574,348,079	+	ATP binding
*TraesCS5A02G378700*	*Os03g0738600*	*OsLOX2*	chr5A	575,705,508	575,709,396	−	Metal ion binding
*TraesCS5A02G374500*	*Os03g0645100*	—	chr5A	572,837,631	572,841,016	+	Catalytic activity
*TraesCS5A02G369500*	—	—	chr5A	569,546,082	569,550,560	−	ATP binding
*TraesCS5A02G373600*	—	—	chr5A	571,679,887	571,683,736	+	GTP binding
*TraesCS5A02G414400*	*Os03g0778100*	—	chr5A	602,355,957	602,357,166	−	Photosystem I
*TraesCS5A02G392300*	*Os03g0754800*	—	chr5A	588,548,724	588,552,987	+	Transmembrane transport
*TraesCS5A02G401700*	*Os03g0764800*	*OsSAPK8*	chr5A	594,570,843	594,577,495	+	ATP binding
*TraesCS5A02G423000*	*Os03g0784700*	*pRRFNR14*	chr5A	608,962,974	608,964,772	+	Chloroplast
*TraesCS5A02G420700*	—	—	chr5A	607,199,244	607,199,355	−	Chloroplast thylakoid membrane
*TraesCS5A02G426100*	*Os03g0787300*	—	chr5A	611,339,186	611,342,238	+	ATP binding
*TraesCS5A02G429000*	*Os03g0791800*	*OsUBC9*	chr5A	613,539,450	613,543,349	+	ATP binding
*TraesCS5A02G424100*	*Os03g0785900*	—	chr5A	609,812,809	609,814,019	+	Glutathione metabolic process
*TraesCS5A02G411200*	—	—	chr5A	599,809,089	599,814,381	−	Electron transport chain
*TraesCS5A02G424400*	*Os03g0786100*	*GLO1*	chr5A	609,862,738	609,865,710	+	Oxidation-reduction process
*TraesCS5B02G356300*	*Os09g0553200*	*OsUgp1*	chr5B	536,045,678	536,052,111	+	Transferase activity

These 18 genes were divided into three categories according to their function. The first category was related to the composition of chloroplasts. *TraesCS5A02G420700* related to chloroplast thylakoid membrane, and *TraesCS5A02G377000* related to chloroplast membrane formation and the homologous gene *TraesCS5A02G423000* of *pRRFNR14* (*Os03g0784700*) in rice involved in the process of chloroplast composition (Aoki and Ida, 1994). The second category was related to eight new genes of chlorophyll photosynthesis, including *TraesCS5A02G414400*, *TraesCS5A02G378700* (*OsLOX*_*2*_), *TraesCS5A02G373600*, *TraesCS5A02G424100*, *TraesCS5A02G376700*, *TraesCS5A02G369500*, *TraesCS5A02G392300*, and *TraesCS5B02G356300* (*OsUgp1*) (Huang et al., 2014; E et al., 2015). These genes participated in photosystem I reaction center subunit III, ATP binding, metal ion binding, and transferase activity. The third kind of genes responded to drought stress by regulating photorespiration, mediating auxin response, and participating in the regulation of ABA signal transduction pathway, such as rice homologous gene *GLO1*, *OsIAA13*/*OsIAA1*, *OsSAPK8*, and *OsUBC9* (Thakur et al., 2001; Zhang et al., 2012; Xu et al., 2013; E et al., 2015). We also identified three novel genes *TraesCS5A02G411200*, *TraesCS5A02G374500*, and *TraesCS5A02G426100* that responded to drought stress by redox reaction, activation of enzyme activity, and ATP binding (Table 4).

## Discussion

### Comparison with Previous Research Results

According to reviews by Gupta et al. (2017, 2020), a total of 82 QTLs controlling chlorophyll content were identified in previous studies. These QTLs were distributed across all 21 chromosomes and explained 2.7–59.1% of the phenotypic variation, but most of these QTLs were different. The reasons could be due to 1) different methods of chlorophyll measurement that cause differences in phenotypic values, e.g., some studies used a spectrophotometer (Zhang et al., 2009b) and others used a chlorophyll meter, leading to differences in QTL analysis results (Bhusal et al., 2018); 2) chlorophyll content is a complex quantitative trait and genes controlling leaf chlorophyll are expressed differently at different developmental stages (Yang D. et al., 2016), and different measurement periods will inevitably lead to different identified genes; 3) due to different types of populations and molecular markers, it is not easy to compare results across different genetic backgrounds.

In this study, 29 QTLs controlling chlorophyll content in flag leaves were located on 12 chromosomes, most of which were A and B genome chromosomes with only three detected in the D genome. Similar results were reported in previous studies (Zhang et al., 2009b; Yang D. et al., 2016). We detected four stably expressed major QTLs on chromosomes 3B (*Qchl.saw-3B.2*), 5A (*Qchl.saw-5A.2* and *Qchl.saw-5A.3*), and 5B (*Qchl.saw-5B.2*), with contribution rates of 5.28–23.25% to the variation in chlorophyll content. These QTLs still need further validation before application in marker-assisted selection (Ahmed et al., 2021).

**FIGURE 3:**
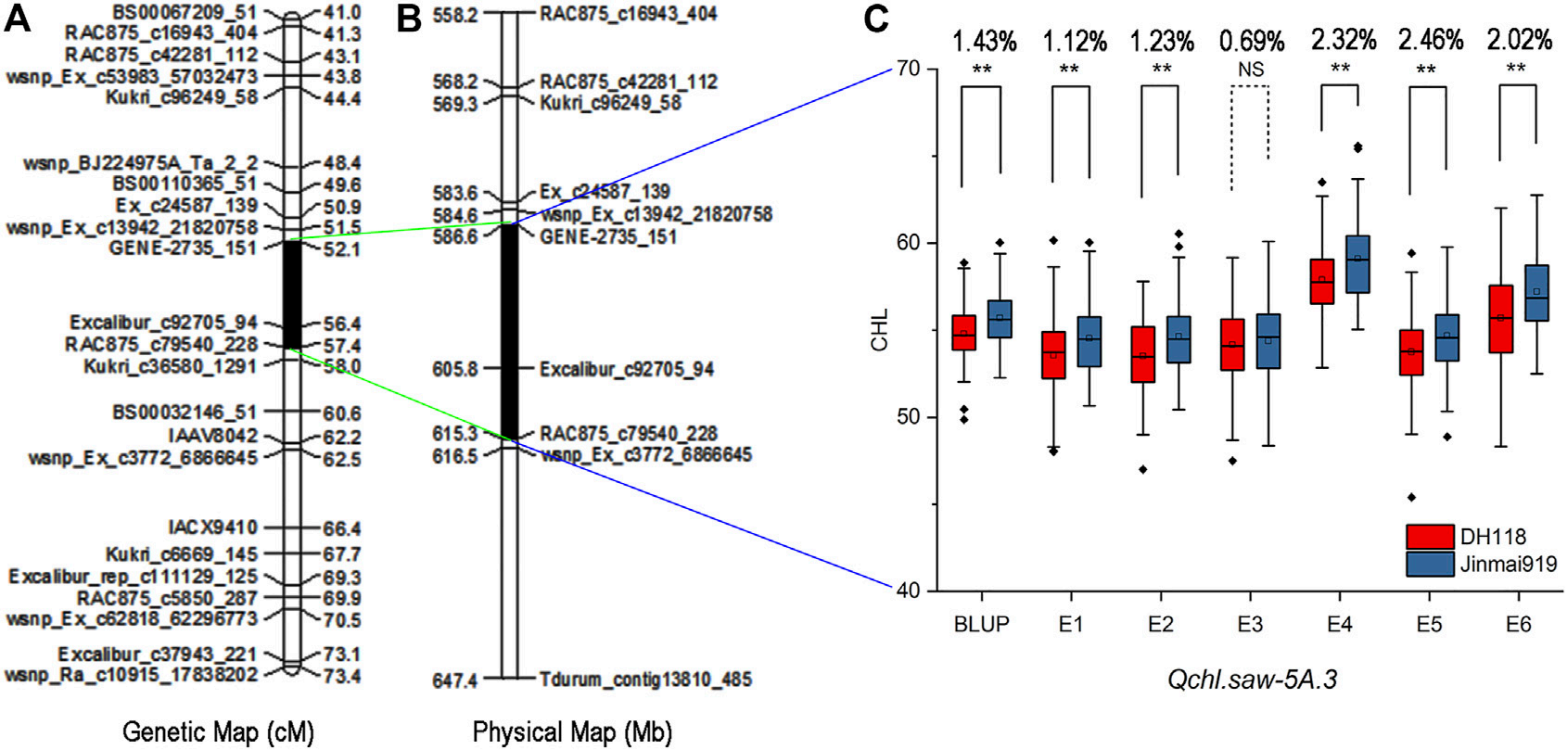
Genetic map of the major QTL *Qchl.saw-5A.3* and its effect. **(A)** Genetic map of *Qchl.saw-5A.3* for chlorophyll content. **(B)** Physical map of flanking markers of *Qchl.saw-5A.3*. **(C)** Effect of the QTL shown as box plots calculated after dividing the DJ population into two classes based on the flanking markers. * and **, *p* <0.05 and *p* <0.01, respectively; NS, not significant.

Fourteen, seven, and nine QTLs for chlorophyll content were located on chromosomes 3B, 5A, and 5B, respectively, in previous studies (Table 5). The three major QTLs controlling chlorophyll content of flag leaves identified in our study were consistent with results of previous studies. The major QTL *Qchl.saw-3B.2* on chromosome 3B was in the interval 52.83–54.75 Mb. Kumar et al. (2010) reported a major QTL *QSg.bhu-3B* for flag leaf senescence in the same region, explaining 17.9% of the variation in stay green phenotypic, and Puttamadanayaka et al. (2020) reported *QChl.iari_3B* that controlled chlorophyll content. The QTLs in our study spanned shorter physical distances and are therefore more conducive for gene cloning. *Qchl.saw-5A.2* was in the range 569.54–582.38 Mb. Puttamadanayaka et al. (2020) reported *QChl.iari_5A* for chlorophyll content spanned by *AX-94531685* (567.52 Mb) and *AX-94726381* (582.96 Mb). In the same region, Wang et al. (2017) detected three major QTLs controlling 1,000-grain weight, and their adjacent markers were *BS00073670_51*, *wsnp_Ex_c1138_2185522*, and *Tdurum_contig71499_211*, respectively. Yang et al. (2019) cloned a *TaGL3-5A* allele that conferred larger grain size based on homology with rice. Many studies have confirmed the high correlation between chlorophyll content and yield-related traits (Zhang et al., 2009b; Vijayalakshmi et al., 2010). Although there was no investigation of yield-related traits in this study, we have co-located QTL/genes for chlorophyll content, 1,000-grain weight, and grain size in the same interval with previous studies and confirmed the correlation between chlorophyll content and yield-related traits. The major QTL *Qchl.saw-5B.2* on chromosome 5B was located in the interval 536.05–536.68 Mb, which coincided with chlorophyll content QTL *Qspad.acs-5B.4* spanned by *Xwmc415* and *Xwmc508* reported by Yang et al. (2016). *Qchl.saw-5A.3* with the strongest genetic effect in our study was in the chromosome 5A interval 586.59–615.30 Mb (Figures 3A–C). Given no previous report of gene for chlorophyll content in this interval, *Qchl.saw-5A.3* is a novel QTL.

**TABLE 5:** Chlorophyll QTLs on chromosomes 3B, 5A, and 5B from previous studies

Chromosome	Left marker	Right marker	Physical interval (Mb)	References
3B	*Xgwm533*	*Xgwm1037*	35.32–77.72	Kumar et al. (2010)
3B	*Xgwm566*	*Xgwm72*	77.72–216.62	Li et al. (2010)
3B	*Xbarc68*	*Xbarc101*	76.13–621.47	Kumar et al. (2012)
3B	*Xwmc326*	—	778.70	Barakat et al. (2015)
3B	*Xgwm264*	*Xgwm566*	68.91–77.72	Awlachew et al. (2016)
3B	*wsnp_Ra_c41135_48426638*	*wsnp_BE497169B_Ta_2_1*	3.41–16.04	Shirdelmoghanloo et al. (2016)
3B	*Xgwm566*	*Xgwm285*	77.72–415.92	Yang B. et al. (2016)
3B	*Xwmc808*	*Xbarc102*	17.57–42.71	Yang D. et al. (2016)
3B	*Xmag3356*	*Xwmc291*	700.81–808.66	Yang D. et al. (2016)
3B	*Xgwm566*	*Xwmc540*	77.72–132.94	Yang D. et al. (2016)
3B	*Xbarc087*	*Xaag/ctc-1*	14.39	Tahmasebi et al. (2017)
3B	*IWB10755*	—	238.82	Maulana et al. (2020)
3B	*Xwmc689*	*Xwmc78*	43.68–201.87	Puttamadanayaka et al. (2020)
3B	*Xgwm340*	*wPt8352*	826.23	Ballesteros et al. (2015)
5A	*Xgwm443*	*P2470-280*	22.71–105.43	Li et al. (2010)
5A	*Xgwm415*	*wPt9452*	692.78	Ballesteros et al. (2015)
5A	*Xbarc122*	—	766.16	Barakat et al. (2015)
5A	*Xgwm154*	*Xgwm156*	21.00–450.16	Yang B. et al. (2016)
5A	*Xwmc410*	*Xgwm595*	678.29–680.07	Yang B. et al. (2016)
5A	*AX-94414339*	*AX-94730618*	556.01–561.11	Puttamadanayaka et al. (2020)
5A	*AX-94531685*	*AX-94726381*	567.52–582.96	Puttamadanayaka et al. (2020)
5B	*Xgwm335*	*Xgwm371*	418.81–447.21	Li et al. (2010)
5B	*Xgwm371*	*Xgwm499*	477.21–477.51	Li et al. (2014)
5B	*Xbcd9*	*Xwg583*	536.05–544.57	Yu et al. (2014)
5B	*Xmag532*	*Xgwm499*	418.81–477.51	Yang D. et al. (2016)
5B	*Xwmc734*	*Xwmc235*	612.87–634.17	Yang D. et al. (2016)
5B	*Xwmc47*	*Xbarc4*	65.95	Bhusal et al. (2018)
5B	*AX-95091073*	*AX-94525,037*	13.73–21.75	Puttamadanayaka et al. (2020)
5B	*Xwmc415*	*Xwmc508*	507.92–654.92	Yang D. et al. (2016)
5B	*Xbarc140*	*Xgdm116*	598.03–618.15	Yang D. et al. (2016)

Spanning markers were used to locate positions in the physical map if the certain markers failed to be located on the physical map. The physical locations of some markers were not available leaving the physical location as a single marker.

### Effect of Environment on Expression of QTL for Chlorophyll Content

Synthesis and degradation of chlorophyll are complex biological processes and regulation likely differs under different water regimes (Yang D. et al., 2016). Under irrigated conditions, higher chlorophyll content could ensure fixation of more photosynthetic assimilates (Zhang et al., 2009b). Under drought stress conditions, stay green is closely related to higher yield (Verma et al., 2004; Thomas and Ougham, 2014). Drought-tolerant genotypes usually have higher chlorophyll content, and chlorophyll degrades more slowly under drought stress (Kumar et al., 2012; Lopes and Reynolds, 2012).

In this study, QTL analysis of chlorophyll content in flag leaves under irrigated and dryland (drought stressed) conditions was made using a RIL population derived from a cross between a variety DH118 recommended for irrigated conditions and drought-resistant variety Jinmai 919. Twelve QTLs were detected under irrigated conditions (E1, E2, E3 and BULP—W), and 18 QTLs were identified under drought stress (E4, E5, E6 and BULP—D) (Table 3). The number of QTLs under drought stress was much more than that under well-watered conditions, showing that environmental stress could induce to express genes originally keeping silent under irrigated conditions to reduce plant damages from environmental stress (Yang et al., 2007; Guo et al., 2008; Vijayalakshmi et al., 2010; Christopher et al., 2018). In addition, it was not difficult to find that there were some differences in QTL mapping data between the well-watered and drought stress, which implied that there were different QTL expression patterns under different water regimes (Yang et al., 2007; Yang B. et al., 2016; Xu et al., 2017; Hassan et al., 2018; Christopher et al., 2021). It also implies that different QTLs should be used for marker-assisted breeding of wheat varieties under irrigated conditions and dryland. For example, the *Qchl.saw-3B.2* detected in this study was not only confirmed to be stably expressed without the influence of genetic background, but also detected under several well-watered conditions, which may be more suitable for molecular marker–assisted selection of varieties under irrigated conditions. In addition, Kumar et al. (2012) and Hassan et al. (2018) considered that the major QTL detected under drought stress may contain genes that contribute to drought resistance and have the application potential to increase yield under drought stress. In our study, three major QTLs (*Qchl.saw-5A.2*, *Qchl.saw-5A.3*, and *Qchl.saw-5B.2*) were detected in drought stress environments. *Qchl.saw-5A.3* could be detected in all drought stress environments (E4, E5, and E6), and the contribution rate to phenotype was 6.04–23.25% (Table 3), which may be more suitable for marker-assisted selection breeding of drought-resistant varieties. In short, this study used high-density chips for QTL mapping, and the SNP and KASP markers of four major QTLs could be applied to the next development of molecular markers under different water conditions.

## Data Availability

The datasets presented in this study can be found in online repositories. The names of the repository/repositories and accession number(s) can be found in the article/Supplementary Material.
