# Supplementary material for: Identification of Genetic Loci Affecting Flag Leaf Chlorophyll in Wheat Grown under Different Water Regimes
Source: Front Genet. 2022 Mar 15;13:832898. doi: 10.3389/fgene.2022.832898 (PMC8965356; doi:10.3389/fgene.2022.832898)
Supplement: Supplementary file 4 [file DataSheet1.docx]

Supplementary Material

# Supplementary Figures and Tables

**Supplementary Figure 1.** Expression profile of candidate genes of *Qchl.saw-3B.2*, *Qchl.saw-5A.2*, *Qchl.saw-5A.3* and *Qchl.saw-5B.2.*

**Supplementary Table S1.** Primer sequences of KASP markers of *Qchl.saw-3B.2*.

**Supplementary Table S2.** Correlation coefficients for chlorophyll content among six environments.

**Supplementary Table S3.** Additive effects of four major QTLs in the RIL population.

**Supplementary Table S4.** QTL section composition of *Qchl.saw-3B.2*, *Qchl.saw-5A.2*, *Qchl.saw-5A.3* and *Qchl.saw-5B.2.* **(See** **Supplementary Table S4.xls)**

**Supplementary Table S5.** Functional annotation and enrichment of chlorophyll content QTL on Chromosome 3B, 5A and 5B **(See** **Supplementary Table S5.xls)**

## Supplementary Figures


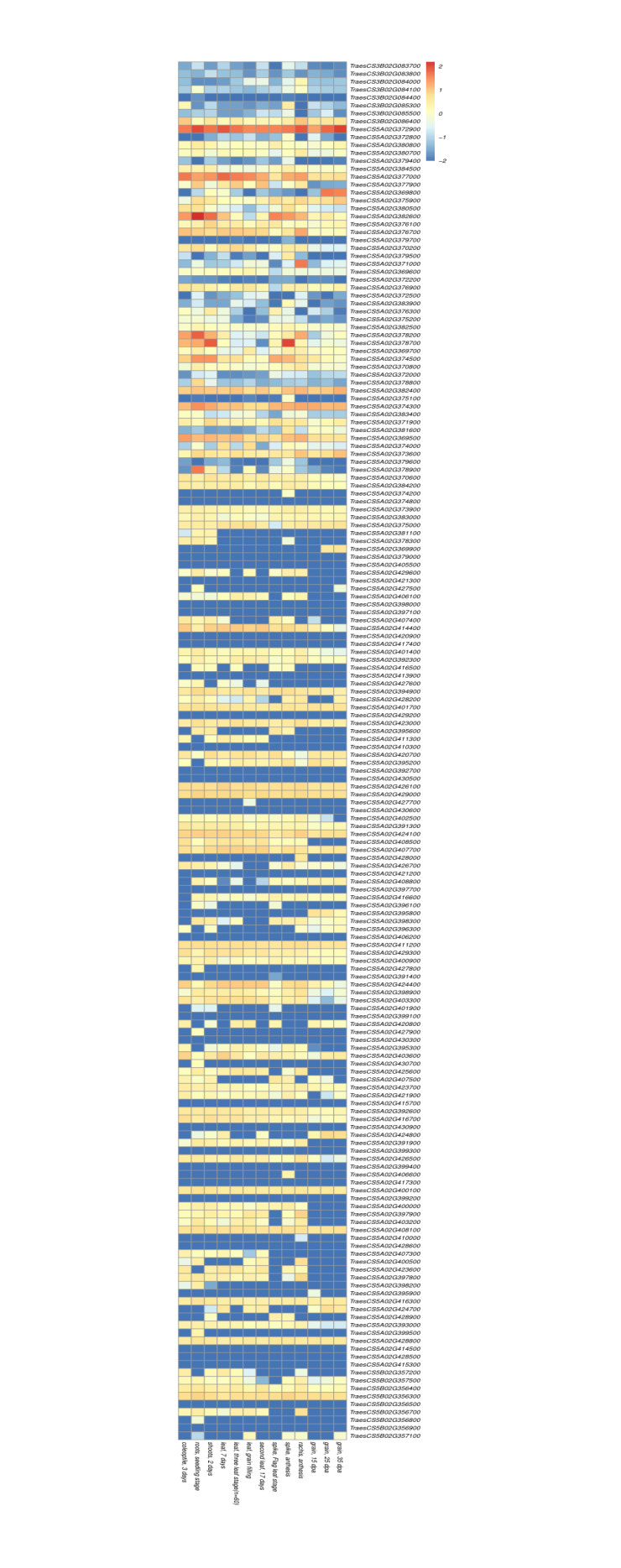


**Supplementary Figure 1.** Expression profile of candidate genes of *Qchl.saw-3B.2*, *Qchl.saw-5A.2*, *Qchl.saw-5A.3* and *Qchl.saw-5B.2.*

## Supplementary Tables

**Supplementary Table S1.** Primer sequences of KASP markers of *Qchl.saw-3B.2*.

| Primer | Sequence |
| --- | --- |
| FAM | TCTCTTCTGTCTAAGACGATGAGA |
| HEX | TCTCTTCTGTCTAAGACGATGAGG |
| Common | TGGTTGCAATGCATGTTGTAT |

**Supplementary Table S2.** Correlation coefficients for chlorophyll content among six environments.

|  | E1 | E2 | E3 | E4 | E5 |
| --- | --- | --- | --- | --- | --- |
| E2 | 0.630** |  |  |  |  |
| E3 | 0.597** | 0.545** |  |  |  |
| E4 | 0.445** | 0.520** | 0.303** |  |  |
| E5 | 0.559** | 0.636** | 0.498** | 0.619** |  |
| E6 | 0.460** | 0.552** | 0.412** | 0.521** | 0.711** |

*Significant at *P* < 0.05; **significant at *P* < 0.01.

**Supplementary Table S3** Additive effects of four major QTLs in the RIL population**.**

| *Qchl.saw-3B.2* | *Qchl.saw-5A.2* | *Qchl.saw-5A.3* | *Qchl.saw-5B.2* | Sample Size | CHL | Difference | Percent (%) |
| --- | --- | --- | --- | --- | --- | --- | --- |
| + | + | + | + | 0 | \ | \ | \ |
| + | + | + | - | 2 | 56.41±0.42b | 3.81 | 7.24 |
| + | - | + | + | 22 | 56.12±1.45ab | 3.52 | 6.69 |
| - | - | + | + | 19 | 56.01±1.85ab | 3.41 | 6.48 |
| + | + | - | + | 20 | 55.71±1.57ab | 3.11 | 5.91 |
| - | + | + | + | 0 | \ | \ | \ |
| + | + | - | - | 18 | 55.25±1.52ab | 2.65 | 5.04 |
| + | - | + | - | 16 | 55.19±1.13ab | 2.59 | 4.92 |
| - | - | + | - | 17 | 54.99±1.25ab | 2.39 | 4.54 |
| - | + | + | - | 1 | 54.91±0.00ab | 2.31 | 4.39 |
| + | - | - | + | 13 | 54.71±1.23ab | 2.11 | 4.01 |
| - | + | - | + | 10 | 54.70±1.10ab | 2.10 | 3.99 |
| - | + | - | - | 12 | 54.32±1.39ab | 1.72 | 3.27 |
| - | - | - | + | 3 | 53.43±0.49ab | 0.83 | 1.58 |
| + | - | - | - | 3 | 53.37±1.00ab | 0.77 | 1.46 |
| - | - | - | - | 5 | 52.60±2.28a | 0.00 | 0.00 |

“+” and “-” represent lines with and without the positive alleles of the target quantitative trait loci based on the flanking markers of the corresponding QTL, respectively. “\” means no data.
